# The *Toxoplasma* Polymorphic Effector GRA15 Mediates Seizure Induction by Modulating Interleukin-1 Signaling in the Brain

**DOI:** 10.1128/mBio.01331-21

**Published:** 2021-06-22

**Authors:** Taylor G. Glausen, Gabriela L. Carrillo, Richard M. Jin, Jon P. Boyle, Jeroen P. J. Saeij, Elizabeth A. Wohlfert, Michael A. Fox, Ira J. Blader

**Affiliations:** a Department of Microbiology and Immunology, SUNY at Buffalo School of Medicine, Buffalo, New York, USA; b Center for Neurobiology Research, Fralin Biomedical Research Institute at Virginia Tech Carilion, Roanoke, Virginia, USA; c Translational Biology, Medicine, and Health Graduate Program, Virginia Tech Roanoke, Roanoke, Virginia, USA; d Department of Biological Sciences, University of Pittsburgh, Pittsburgh, Pennsylvania, USA; e Department of Pathology, Microbiology, and Immunology, University of California, Davis, Davis, California, USA; f School of Neuroscience, Virginia Tech, Blacksburg, Virginia, USA; g Department of Biological Sciences, Virginia Tech, Blacksburg, Virginia, USA; h Department of Pediatrics, Virginia Tech Carilion School of Medicine, Roanoke, Virginia, USA; Albert Einstein College of Medicine

**Keywords:** *Toxoplasma gondii*, encephalitis, host-parasite relationship, neuroimmunology

## Abstract

Toxoplasmic encephalitis can develop in individuals infected with the protozoan parasite Toxoplasma gondii and is typified by parasite replication and inflammation within the brain. Patients often present with seizures, but the parasite genes and host pathways involved in seizure development and/or propagation are unknown. We previously reported that seizure induction in *Toxoplasma*-infected mice is parasite strain dependent. Using quantitative trait locus mapping, we identify four loci in the *Toxoplasma* genome that potentially correlate with seizure development. In one locus, we identify the polymorphic virulence factor, GRA15, as a *Toxoplasma* gene associated with onset of seizures. GRA15 was previously shown to regulate host NF-κB-dependent gene expression during acute infections, and we demonstrate a similar role for GRA15 in brains of toxoplasmic encephalitic mice. GRA15 is important for increased expression of interleukin 1 beta (IL-1β) and other IL-1 pathway host genes, which is significant since IL-1 signaling is involved in onset of seizures. Inhibiting IL-1 receptor signaling reduced seizure severity in *Toxoplasma*-infected mice. These data reveal one mechanism by which seizures are induced during toxoplasmic encephalitis.

## INTRODUCTION

Toxoplasma gondii is an obligate intracellular protozoan parasite that infects approximately one-quarter of the world’s population (1, 2). Infection occurs via consumption of parasites, either within tissue cysts or oocysts, in contaminated food and water sources. Asymptomatic in otherwise healthy individuals, *Toxoplasma* can cause serious and life-threatening disease in developing fetuses and immunosuppressed patients. Both patient groups can develop encephalitis that often presents with neurological complications, including seizures (3). How seizures develop during toxoplasmic encephalitis is largely unknown, although recent work has revealed that *Toxoplasma* enhances excitatory and reduces inhibitory neurotransmission by altering neuronal synapse structure and function (4–6).

*Toxoplasma* has a unique population structure with three clonal lineages that predominate throughout North America and Europe. The most distinguishing trait of these three lineages is their virulence in mice. Strains belonging to the type I lineage kill mice within 10 days postinfection, with a lethal dose (LD) of 1 parasite (7). Type II parasites are moderately virulent, with a 50% LD (LD_50_) of 10^3^ parasites, and type III parasites are significantly less virulent, with an LD_50_ of 10^5^ parasites (7). Quantitative trait locus (QTL) mapping using progeny from genetic crosses between these three strain types has led to the identification of 5 virulence loci (VIR1 to VIR5). A pseudokinase, ROP5 (identified as VIR1), is the major polymorphic *Toxoplasma* virulence factor and works in concert with a serine/threonine kinase, ROP18 (identified as VIR3), to prevent a family of interferon-inducible GTPases from triggering degradation of the parasitophorous vacuole within which *Toxoplasma* replicates (8–13). ROP16 (VIR4) and another polymorphic factor, GRA15, function by regulating the host cell transcription factors STAT3/6 and NF-κB, respectively (14–16), thereby impacting polarization of a host’s immune response to be either proinflammatory (GRA15-NF-κB) or anti-inflammatory (ROP16-STAT3/6). During the acute phase of the infection, type II parasites promote proinflammatory cytokine expression because they express functional GRA15 and less active ROP16 gene products (17, 18). But a role for GRA15 (and, to a lesser extent, ROP16) has primarily been examined in the context of acute infections, and therefore, it is unclear how it impacts tissues such as the brain during long-term chronic infections or in a host suffering from toxoplasmosis due to a reactivated infection (14, 17–19).

Previously, we reported that *Toxoplasma* induces seizures in a strain type-dependent manner. Specifically, type II strain-infected mice develop both spontaneous and drug-induced seizures, whereas type III strain-infected mice do not (4). These data suggest the involvement of one or more genetically encoded polymorphic factor(s). Using progeny from a cross between type II and III strain parasites, QTL mapping was employed to identify *Toxoplasma* seizure-linked genes (SLGs). Four SLG loci were identified in the *Toxoplasma* genome, and we determined that one of these contained GRA15. Furthermore, we demonstrate that GRA15 acts to create a proconvulsant state by increasing interleukin 1 (IL-1) signaling.

## RESULTS

### Quantitative trait locus mapping identifies genomic loci associated with seizure susceptibility.

Seizures induced by low doses of the GABA antagonist pentylenetetrazole (PTZ) are triggered more rapidly and are more severe in mice infected with type II strain parasites than those infected with type III strains (4). These data suggest the involvement of one or more genetically encoded polymorphic seizure-linked genes (SLGs) that we sought to identify by QTL mapping. Forty progeny strains have been isolated from crosses between types II (ME49) and III (CEP) strains, and virulence varies considerably between the progeny, with some being significantly more or less virulent than either parental strain (11, 20). Because we assess seizures 30 days postinfection, hypervirulent progeny strains were excluded from our assays because they kill mice within 12 days. Thus, we were limited to testing responses to PTZ of only 24 progeny strains, which reduced overall statistical power of our analyses.

**FIG 1:**
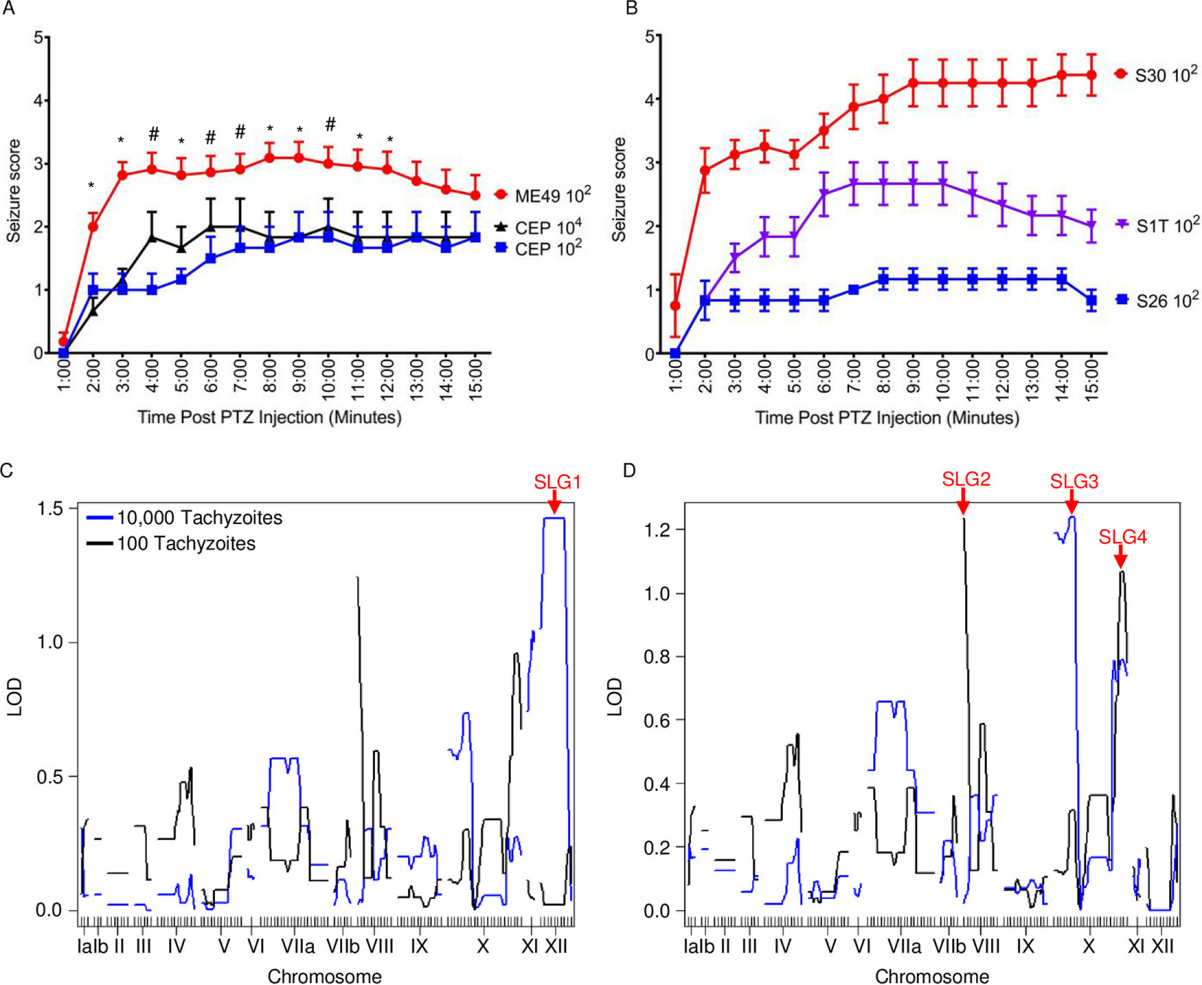
Identification of SLG QTLs. (A) PTZ-induced seizures were measured in C57BL/6 mice infected with ME49 (type II) or CEP (type III) with 100 or 10,000 (CEP only) tachyzoites for 30 days. *n* = 6 to 9 mice per infection dose. #, *P* ≤ 0.05 ME49 versus CEP 100 tachyzoite dose; *, *P* ≤ 0.05 ME49 versus either CEP dose; Student's *t* test. (B) Examples of progeny strains presenting with strong (S30), medium (S1T), and weak (S26) PTZ-induced seizures. Shown are means ± SEM for 6 to 9 mice per strain. (C and D) QTL mapping of seizure phenotypes for progeny infected with 10,000 (blue line) or 100 (black line) tachyzoites. Seizure-linked genes (SLGs) loci are labeled in red. (C) Primary scan revealed a peak on chromosome XII (SLG 1), which correlates with the location of ROP5. (D) Secondary scan performed using SLG1 as a covariant. In panels C and D, *P* = 0.05 at LOD of 2.88 (high dose) and 2.62 (low dose) and *P* = 0.1 at LOD of 2.29 (high dose) and 2.23 (low dose).

Similar to reference 11, mice were intraperitoneally infected with 100 or 10,000 tachyzoites of each progeny or the parental ME49 and CEP strains (21) (note that mice were only assayed with 100 ME49 tachyzoites because high-dose infections were lethal [data not shown]). Mice were challenged with PTZ 30 days later and seizure scores recorded every minute for 15 min. As previously reported (4), mice infected with parental type II ME49 parasites developed more rapid and severe seizures than those infected with type III CEP strain parasites (Fig. 1A). Seizure phenotypes in mice infected with progeny strains were more wide-ranging, with some strains producing more severe seizures than type II strains and some with less severe seizures than type III strains (Fig. 1B and Fig. S1 in the supplemental material).

10.1128/mBio.01331-21.1 FIG S1 Seizure response of 24 progeny strains. (A to X) Seizure responses were recorded at 30 dpi with 100 or 10,000 tachyzoites of each progeny strain, with the exception of CL12 in which infections with 10,000 tachyzoites were lethal during acute infections. *n* = 3 to 9 mice per dose. Data represent mean ± SEM. Download FIG S1, TIF file, 1.9 MB. Copyright © 2021 Glausen et al. 2021 Glausen et al. https://creativecommons.org/licenses/by/4.0/ This content is distributed under the terms of the Creative Commons Attribution 4.0 International license.

The PTZ data were binned to determine the percentage of time that each seizure score was recorded following PTZ administration. Based upon binned data of the parental strains, we defined a severe seizure phenotype for each infectious dose as follows: (i) seizure scores of ≥3 for at least 15% of the trial for mice infected with 100 tachyzoites, and (ii) seizure scores of ≥3 for at least 30% of the trial for mice infected with 10,000 tachyzoites (Table S1). Strains that generated a severe seizure phenotype were denoted as “2” (type II ME49-like), while those with scores below the threshold were denoted as “3” (type III CEP-like). Binned seizure phenotype data from the progeny were used for QTL analysis to identify SLG loci in the *Toxoplasma* genome. A predominate peak (SLG1) was noted on chromosome XII in the high-dose seizure scan (Fig. 1C). The ROP5 gene is located within this QTL, and because ROP5 has such a profound effect on virulence (11–13), we hypothesized that ROP5 was likely involved in seizure production. We, therefore, adapted the approach of reference 11 and fixed SLG1 as a covariate for a second QTL scan. The second scan revealed 3 additional SLG loci (Fig. 1D).

10.1128/mBio.01331-21.6 TABLE S1 Seizure phenotyping of 24 progeny strains. Mice infected with parental or progeny strains for 30 days were injected i.p. with PTZ (40 mg/kg), and seizure scores were recorded for 15 minutes. Scores were binned to create a percent time seizing variable, indicating percentage of time with a seizure score of ≥3. Seizure phenotypes were defined as those with scores ≥3 for at least 15% of the trial (100-tachyzoits infection) or at least 30% of the trial (10,000-tachyzoite infection). ME49 phenotype is coded as a 2, whereas CEP phenotype is coded as a 3. ND, not determined. Download Table S1, XLSX file, 0.01 MB. Copyright © 2021 Glausen et al. 2021 Glausen et al. https://creativecommons.org/licenses/by/4.0/ This content is distributed under the terms of the Creative Commons Attribution 4.0 International license.

We did note, however, that, logarithm of odds (LOD) scores for each QTL were below statistical significance, which could be due to the limited number of progeny that were tested. To address this, QTL scans using virulence data from reference 11 for the 24 strains tested in this study were simulated in which ROP5 was used as a covariate (secondary scan) or not (primary scan). As expected, a significant QTL peak was identified in the chromosomal region associated with ROP5 (Fig. S2). Reflective of the low number of progeny that were analyzed, we similarly observed that LOD scores corresponding to peaks associated with other VIR loci (VIR2 to VIR4 but not VIR5) were no longer statistically significant.

10.1128/mBio.01331-21.2 FIG S2 Virulence QTL analysis of the 24 progeny strains tested for seizure responses. Using virulence phenotype data from reference 11, virulence QTL scans were performed for only the 24 progeny used in this study from Fig. 1. Phenotypes (as denoted by reference 11) were defined by virulence at high-dose (blue) and low-dose (black) infections as well as a binary phenotype regardless of dose (red). (A) Primary scan. (B) Secondary scan in which the ROP5 peak on chromosome XII was used as a covariate. All five virulence QTLs (VIR1 to VIR5) from reference 8 are labeled in red. For the primary scan, a *P* value of 0.05 is a LOD of 2.88 for binary, 2.85 for high dose, and 1.89 for low dose. For the secondary scan, a *P* value of 0.05 is a LOD of 2.89 for binary, 2.77 for high dose, and 1.89 for low dose. Download FIG S2, TIF file, 0.3 MB. Copyright © 2021 Glausen et al. 2021 Glausen et al. https://creativecommons.org/licenses/by/4.0/ This content is distributed under the terms of the Creative Commons Attribution 4.0 International license.

Next, we sought to determine whether seizures and morbidity were linked traits by comparing weight loss, which is an indicator of *Toxoplasma*-induced morbidity in mice (22). We found that while there was a general correlation between seizure severity and morbidity (*P* = 0.37; McNemar chi-square test), the two did not absolutely cosegregate (Table S2). We also examined whether cyst burdens cosegregated with seizure susceptibility in *Toxoplasma*-infected mice. Cysts were quantified in coronal brain sections obtained from sections that fell within a 3-mm window along the anterior-posterior axis of the brain (−3 mm to 0 mm from the anatomical landmark bregma). If ≥5 cysts were detected in 12 serial coronal sections they were denoted ME49-like (“2”), and CEP-like (“3”) if <5 were detected. In contrast to weight loss, we observed that cyst burden and seizure phenotypes did not correlate with each other (*P* = 0.0133; McNemar chi-square test; Table S2). This is not surprising given that tachyzoites rather than cysts underlie onset of toxoplasmic encephalitis (23). Together, these data indicate that susceptibility to *Toxoplasma*-induced seizures is likely dependent on multiple polymorphic *Toxoplasma* genes.

10.1128/mBio.01331-21.7 TABLE S2 Seizures, cysts, weight loss, and GAD67 mislocalization phenotypes. Seizure scores, brain cyst burdens, weight loss, and GAD67 mislocalization were assessed for the parental and progeny strains. ME49 phenotype is coded as a 2, whereas CEP phenotype is coded as a 3. Weight loss was calculated by the weight gained or lost relative to day 0 of infection, and data were combined from both infectious doses. Strains which induced a weight loss of ≥7.5% were defined as an ME49 phenotype (2). Cyst burden was quantified by counting cysts stained with Dolichos biflorus agglutinin in paraformaldehyde fixed tissue samples of infected mice. An ME49 phenotype for cyst burden is defined by greater than 5 cysts found per 8 brain sections. GAD67 mislocalization was observed only at the 100-tachyzoite infection dose. Mislocalization was determined by staining brain sections with a GAD67 antibody and quantifying mislocalization as a ratio of GAD67 immunoreactivity within the stratum pyramidalis to surrounding areas. A low ratio, and thereby greater mislocalization, is defined as an ME49 phenotype. ND, not determined. Download Table S2, XLSX file, 0.01 MB. Copyright © 2021 Glausen et al. 2021 Glausen et al. https://creativecommons.org/licenses/by/4.0/ This content is distributed under the terms of the Creative Commons Attribution 4.0 International license.

### GRA15 is SLG4.

**FIG 2:**
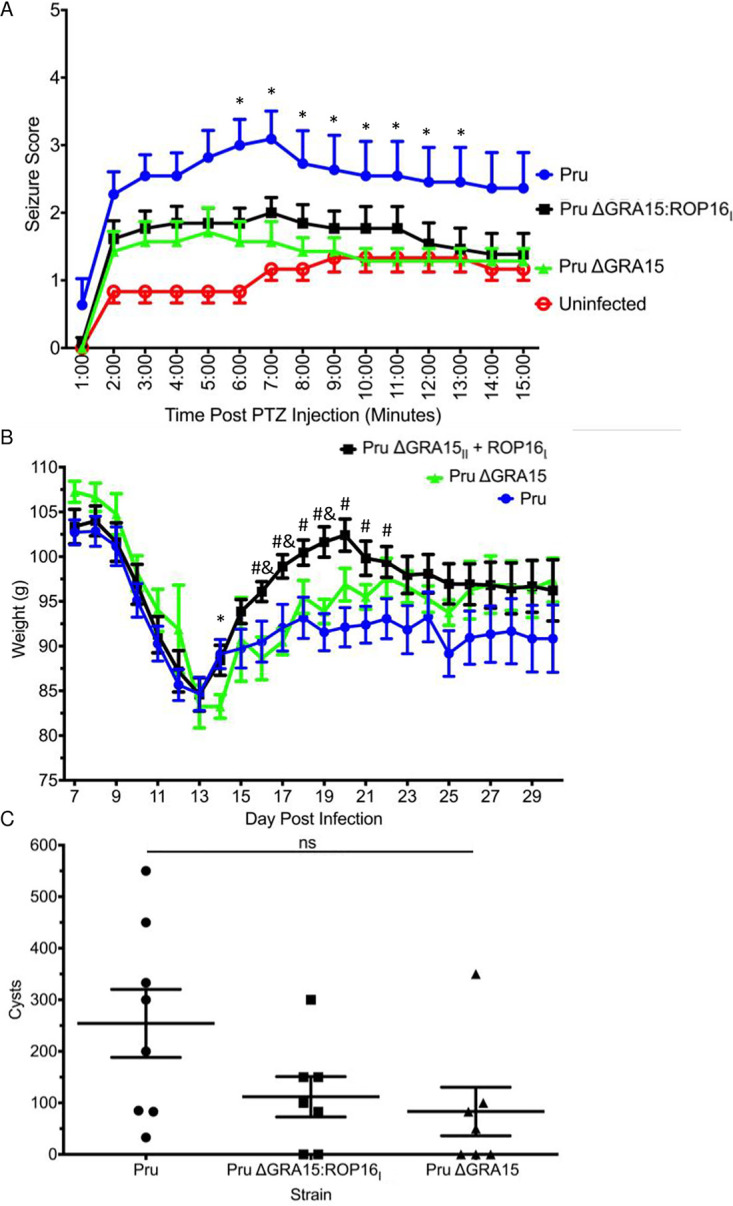
Identification of GRA15 as SLG4. Seizure responses (at 30 dpi) (A), weight loss (B), and cyst burdens (at 30 dpi) (C) of mice infected with Pru (*n* = 11), Pru ΔGRA15 (*n* = 7), Pru ΔGRA15:ROP16_I_ (*n* = 13) (100 tachyzoites/mouse), or uninfected (*n* = 6). Data represents mean ± SEM. *, *P* ≤ 0.05 between Pru and Pru ΔGRA15; #, *P* ≤ 0.05 between Pru and Pru ΔGRA15:ROP16_I_; $, *P* ≤ 0.05 between Pru and both Pru ΔGRA15 and Pru ΔGRA15:ROP16_I_; &, *P* ≤ 0.05 between Pru ΔGRA15 and Pru ΔGRA15:ROP16_I_; ns, not statistically different. Mann-Whitney U test.

We hypothesize that SLGs are polymorphic genes that encode proteins containing signal sequences and/or transmembrane domains to facilitate host-pathogen interactions. Genomic loci of each SLG locus were examined using these filtering criteria to identify candidate seizure-inducing genes (Table S3). SLG2 contains 102 candidate genes, but the predicted function of these genes did not readily identify a seizure-linked gene. SLG3 is located in the same region as VIR2, which contains 59 candidate genes, including the recently identified GRA35, which regulates host cell pyroptosis in Lewis rats, and GRA2, an intravacuolar network protein important for cyst development (24, 25). On chromosome X, SLG4 contained 26 candidate genes, including GRA15 and GRA6, which is another intravacuolar network protein important for cyst development (25). A lack of linkage between cyst and seizure phenotypes suggested that dense granule genes involved in cyst development/maintenance are likely not associated with seizures in *Toxoplasma*-infected mice. GRA15 encodes a polymorphic dense granule protein that, in *Toxoplasma*-infected cells, localizes to the parasitophorous vacuolar membrane and promotes proinflammatory gene expression by promoting the host transcription factor, NF-κB, to remain active during infection (14). Because NF-κB activity is associated with seizures (26, 27), we focused on GRA15 to determine whether it is a bona fide SLG. C57BL/6 mice were infected for 30 days with either wild-type type II Pru or PruΔGRA15 strain parasites, and PTZ-induced seizure responses were recorded. We found that seizures were markedly reduced in PruΔGRA15 strain-infected mice (Fig. 2A).

10.1128/mBio.01331-21.8 TABLE S3 List of genes located within SLG 1, SLG2, SLG3, and SLG4. Gene lists were compiled for each SLG and contain the gene name, product description, and gene name, if present in https://toxodb.org/toxo/app, for every gene within the locus. Genes were color-coded red if they contain ≥1 nonsynonymous single-nucleotide polymorphism (SNP). Genes in gray boxes contain a transmembrane domain and/or a signal sequence. Download Table S3, XLSX file, 0.04 MB. Copyright © 2021 Glausen et al. 2021 Glausen et al. https://creativecommons.org/licenses/by/4.0/ This content is distributed under the terms of the Creative Commons Attribution 4.0 International license.

We were precluded from using a complemented PruΔGRA15:GRA15 strain because the complemented strain was hypervirulent and a significant fraction of infected mice succumbed during the acute phase of the infection (Fig. S3). We therefore used an independently generated GRA15 knockout strain, PruΔGRA15:ROP16_I_, which was also engineered to express the more active allele of the ROP16 rhoptry kinase that functions in opposition to GRA15 by upregulating anti-inflammatory host gene expression (17). Similar to PruΔGRA15, PTZ-induced seizure responses in mice infected with PruΔGRA15:ROP16_I_ were significantly reduced (Fig. 2A). Mice infected with PruΔGRA15:ROP16_I_ lost similar amounts of weight during the acute stage of the infection as those infected with wild-type parasites (Fig. 2B), indicating, as previously reported (14), that GRA15 does not dramatically impact morbidity. While these mice regained weight more quickly than those infected with wild-type parasites, these differences were not significantly different at the time of PTZ administration. Similarly, mice infected with PruΔGRA15 parasites lost weight at similar rates as those infected with wild-type parasites. And, although we noted a trend of increased cyst burdens in brains of mice infected with wild-type parasites, the differences were not statistically significant (Fig. 2C), which is consistent with previous studies (28). Finally, GRA15 expression is significantly reduced in cysts during a chronic infection (29–31). Together, these data indicate that GRA15 is most likely the seizure-linked gene within the SLG4 QTL and that its ability to promote seizures is most likely not a direct consequence of differences in morbidity or brain cyst burdens. Because seizure responses were similar between PruΔGRA15 and PruΔGRA15:ROP16_I_ strain parasites, and since ROP16 did not map to an SLG despite the progeny we tested expressing either of the two ROP16 alleles, unless otherwise noted, the remaining experiments were performed using PruΔGRA15:ROP16_I_ strain parasites to ensure that we were including the potential impact of the active ROP16 allele on toxoplasmic encephalitic inflammatory responses.

10.1128/mBio.01331-21.3 FIG S3 PruΔGRA15:GRA15 is hypervirulent during acute-stage infections. C57BL/6 mice were infected with 100 tachyzoites of Pru (*n* = 27), PruΔGRA15 (*n* = 8), PruΔGRA15:ROP16_I_ (*n* = 23), or PruΔGRA15:GRA15 (*n* = 16) and monitored daily. Download FIG S3, TIF file, 0.4 MB. Copyright © 2021 Glausen et al. 2021 Glausen et al. https://creativecommons.org/licenses/by/4.0/ This content is distributed under the terms of the Creative Commons Attribution 4.0 International license.

### GRA15 promotes pro-inflammatory responses in brains of *Toxoplasma*-infected mice.

**FIG 3:**
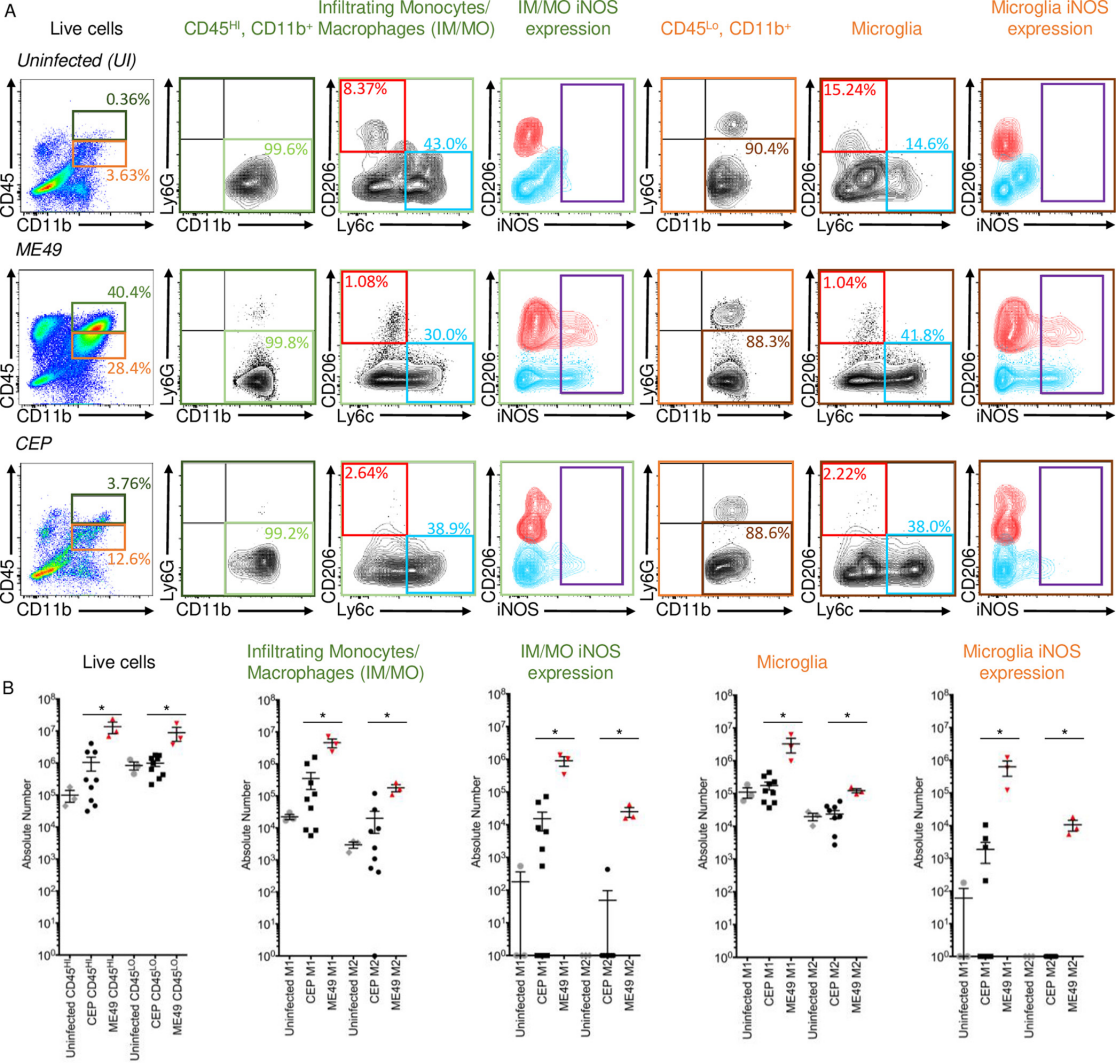
Microglia and inflammatory monocyte/macrophage polarization in *Toxoplasma*-infected brains. (A) Mice were mock infected (*n* = 3) or infected with 100 CEP (*n* = 9) or ME49 (*n* = 3) strain tachyzoites. After 30 days, their brains were harvested and microglia and IM/MO analyzed by flow cytometry. Live single cells were identified by size gating and live/dead cell staining. Microglia and IM/MO were defined as CD45^lo^ (orange box) or CD45^hi^ (dark green box), respectively (first column). Monocytic cells were gated from this population as CD11b^+^/Ly6G^−^ cells (second and fifth columns). M1/M2 polarization was assessed for each cell type using CD206 (M2; red box) and Ly6C (M1; blue box) staining (third and sixth columns). iNOS expression was examined in each cell type (purple box; fourth and seventh columns). Shown are representative plots of 3 independent experiments. (B) Quantification of absolute cell numbers for prominent cell populations identified in panel A. Shown are means ± SEM of 3 independent experiments. *, *P* ≤ 0.05; Mann-Whitney U test.

During acute-stage infections, GRA15 expressed by type II strain parasites promotes M1 macrophage polarization, while type III strains utilize ROP16 to stimulate M2 polarization (17). To determine whether GRA15 and ROP16 have similar functions in the brain at 30 days postinfection, we first compared inflammatory responses between ME49- and CEP-infected brains. Thus, mice were mock or parasite infected, and 30 days later, their brains were harvested and analyzed by flow cytometry (Fig. 3). Similar to references 5 and 32, monocytic cells were defined as CD45-positive (CD45^+^)/CD11b^+^/Ly6G-negative (Ly6G^−^) cells with microglia (MG) as CD45^lo^ and infiltrating monocytes/macrophages (IM/MO) as CD45^hi^ (33). Finally, M1 cells were defined as Ly6C^+^/CD206^−^ and M2 cells as Ly6C^−^/CD206^+^ (34). Infections with type II strain parasites led to increased numbers of both microglia and IM/MO, and the majority of these were Ly6C^+^ (Fig. 3). We next assessed the activation state of these cells by examining inducible nitric oxide synthase (iNOS) expression and found that a significant number of the M1 cells were iNOS^+^. Interestingly, a significant number of CD206^+^ microglia were also iNOS^+^, suggesting that they are similar to polyfunctional macrophages described in reference 35. CEP infection led to increased numbers of microglia but not to the same extent as ME49. Moreover, few microglia from type III strain-infected mice were iNOS^+^, suggesting that the vast majority were not functionally active.

**FIG 4:**
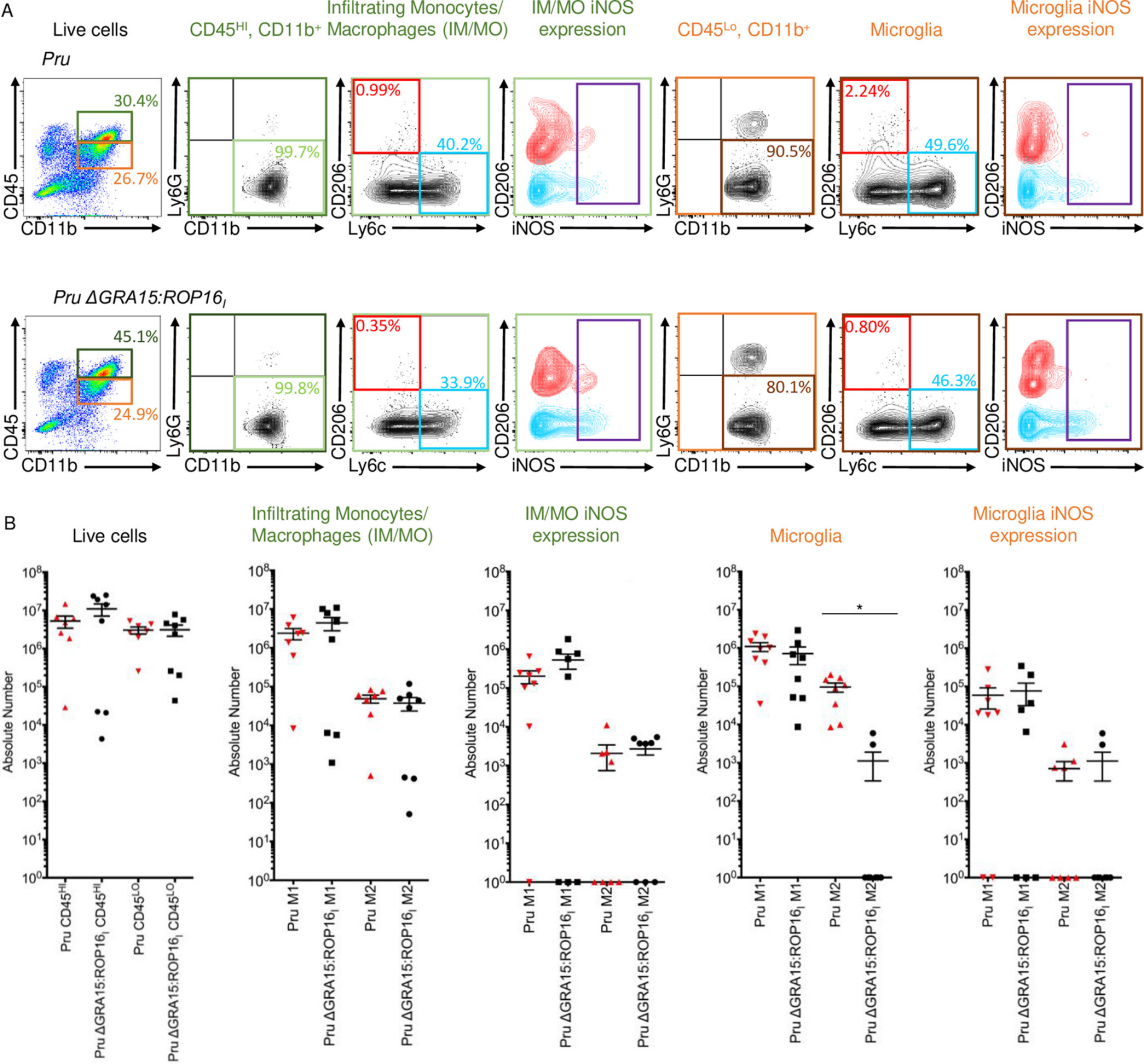
GRA15 is dispensable for microglia and IM/MO expansion in *Toxoplasma*-infected brains. (A) Microglia and IM/MO were quantified in mice that were either mock infected (*n* = 3) or infected with Pru (*n* = 7) or PruΔGRA15:ROP16_I_ (*n* = 8) for 30 days. The cells were identified and analyzed as in Fig. 3. (B) Quantification of absolute number of cells per sample for cell populations of interest. Shown are means ± SEM. *, *P* ≤ 0.05; Mann-Whitney U test.

We next assessed whether GRA15 was important for the increased numbers of activated microglia and monocytes in brains of type II strain-infected mice. Thus, mice were infected for 30 days with wild type (WT) or PruΔGRA15:ROP16_I_ and brains harvested and processed for flow cytometry as described above. We found that total numbers of microglia and IM/MO were not significantly different (Fig. 4). Moreover, no significant differences in M1-polarized cells were noted, although an increase in numbers of M2-polarized microglia was identified (Fig. 4B). This indicates that GRA15 does not have a significant role in the increased numbers of IM/MO and microglia within brains of *Toxoplasma*-infected mice.

### GRA15 modulates IL-1 pathway gene expression in *Toxoplasma*-infected brains.

**FIG 5:**
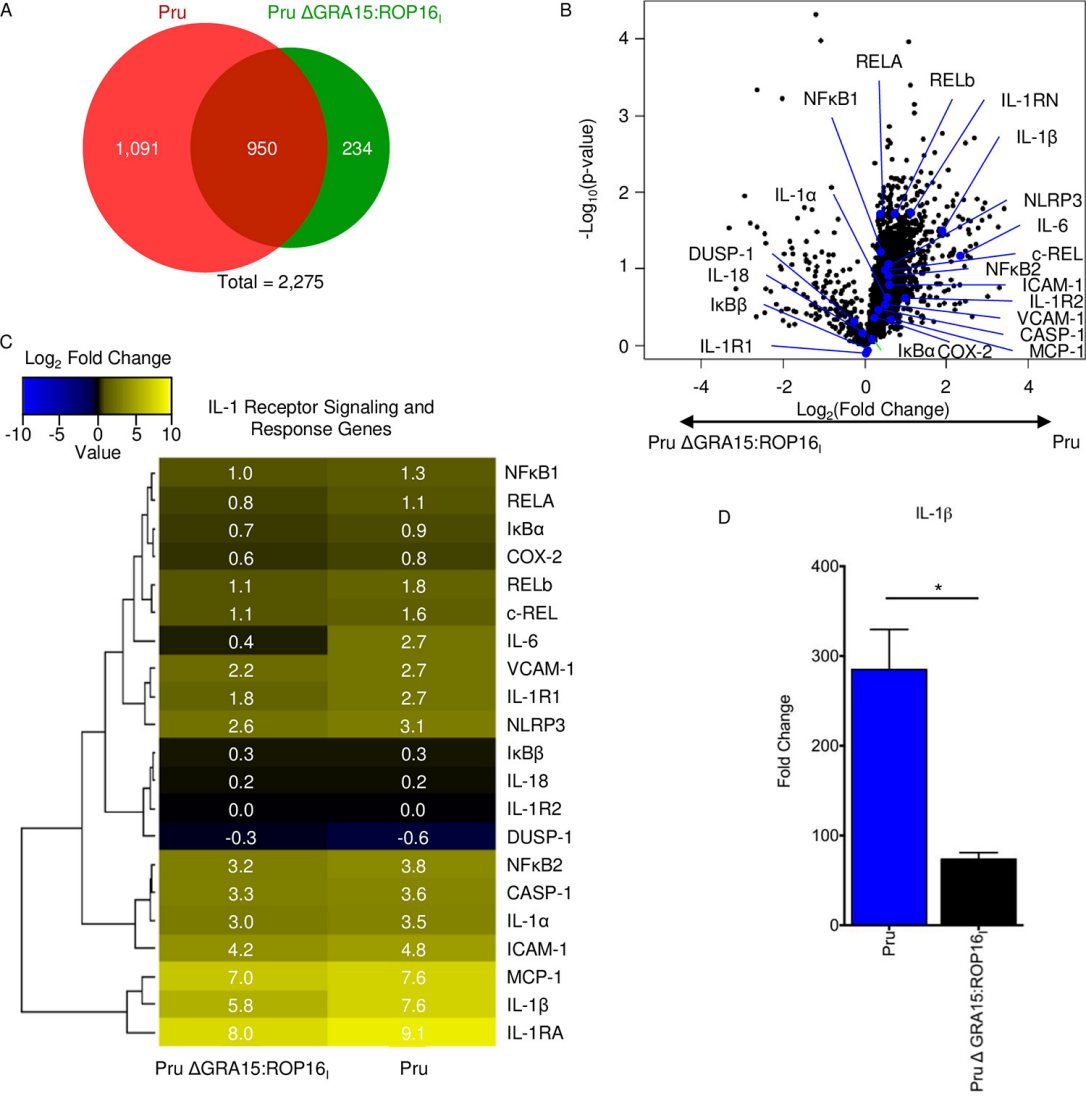
GRA15 regulates IL-1 pathway gene expression in *Toxoplasma*-infected brains. (A) Hippocampal total RNA was isolated from mice 30 days after they were either mock infected (*n* = 3) or infected with Pru (*n* = 3) or PruΔGRA15:ROP16_I_ (*n* = 3) strain parasites. RNA was converted to cDNA and analyzed by RNA sequencing. Significantly upregulated genes were defined by a *P* value of ≤0.05 and a fold change of ≥2. Significantly downregulated genes were defined by a *P* value of ≤0.05 and a fold change of ≤0.5. Student’s *t* tests were used to test for statistical significance. (B) Volcano plot of the significantly up- or downregulated genes from panel A following abundance testing to identify host transcripts whose levels are dependent on GRA15 expression. IL-1 pathway genes that were significantly modulated by GRA15 are highlighted by blue dots and fonts. (C) Heat map of the log_2_ fold change of IL-1 pathway-associated genes. (D) Quantitative RT-PCR was used to measure IL-1β gene expression levels. Shown are means ± SEM. *, *P* ≤ 0.05; Student's *t* test.

Since GRA15 did not significantly impact numbers of microglia and IM/MO cells within the brain, high-throughput RNA sequencing analysis (RNA-seq) was next used to identify genes differentially regulated by GRA15 in murine brains 30 days postinfection. Thus, total RNA was purified from hippocampi isolated from mice 30 days after they were either mock infected or infected with wild-type or PruΔGRA15:ROP16_I_ strain parasites. Hippocampal RNA was used because defects in synaptic connectivity have been identified in hippocampi of *Toxoplasma*-infected mice (4, 36). We found that 2,275 genes were significantly up- or downregulated at least 2-fold by either strain (Fig. 5A and B). Of these, ∼48% were specifically modulated by wild-type strain parasites, while ∼10% were only modulated by PruΔGRA15:ROP16_I_ parasites.

Gene ontology pathway analysis of the differentially expressed genes revealed that loss of GRA15 had a significant impact on immune response-related pathways (7 of top 10 differentially modulated pathways), revealing that an overall importance of GRA15 is coordinating host immunity (Table S4). IL-1β was found in the gene lists for 4 of these 7 pathways, which was of interest because IL-1β signaling increases seizure susceptibility and pharmacological inhibition of this pathway reduces seizure severity (37–39). Further analysis of the RNA-seq data indicated that other genes associated with IL-1 signaling were among the host genes whose expression was impacted by GRA15 (Fig. 5B and C). These included genes whose products are involved in IL-1 expression and signaling (e.g., IL-1β, IL-1α, IL-1RA, CASP1, and NLRP3) as well as IL-1 target genes (21), which is consistent with previous work showing that GRA15 regulates expression of these genes (40, 41). Because IL-1β was significantly more strongly upregulated than IL-1α, we focused on analyzing IL-1β expression in *Toxoplasma*-infected brains and confirmed the RNA-seq data by reverse transcriptase PCR (RT-PCR) (Fig. 5D).

10.1128/mBio.01331-21.9 TABLE S4 List of gene ontology (GO) term pathways differentially regulated by GRA15. Download Table S4, XLSX file, 0.03 MB. Copyright © 2021 Glausen et al. 2021 Glausen et al. https://creativecommons.org/licenses/by/4.0/ This content is distributed under the terms of the Creative Commons Attribution 4.0 International license.

**FIG 6:**
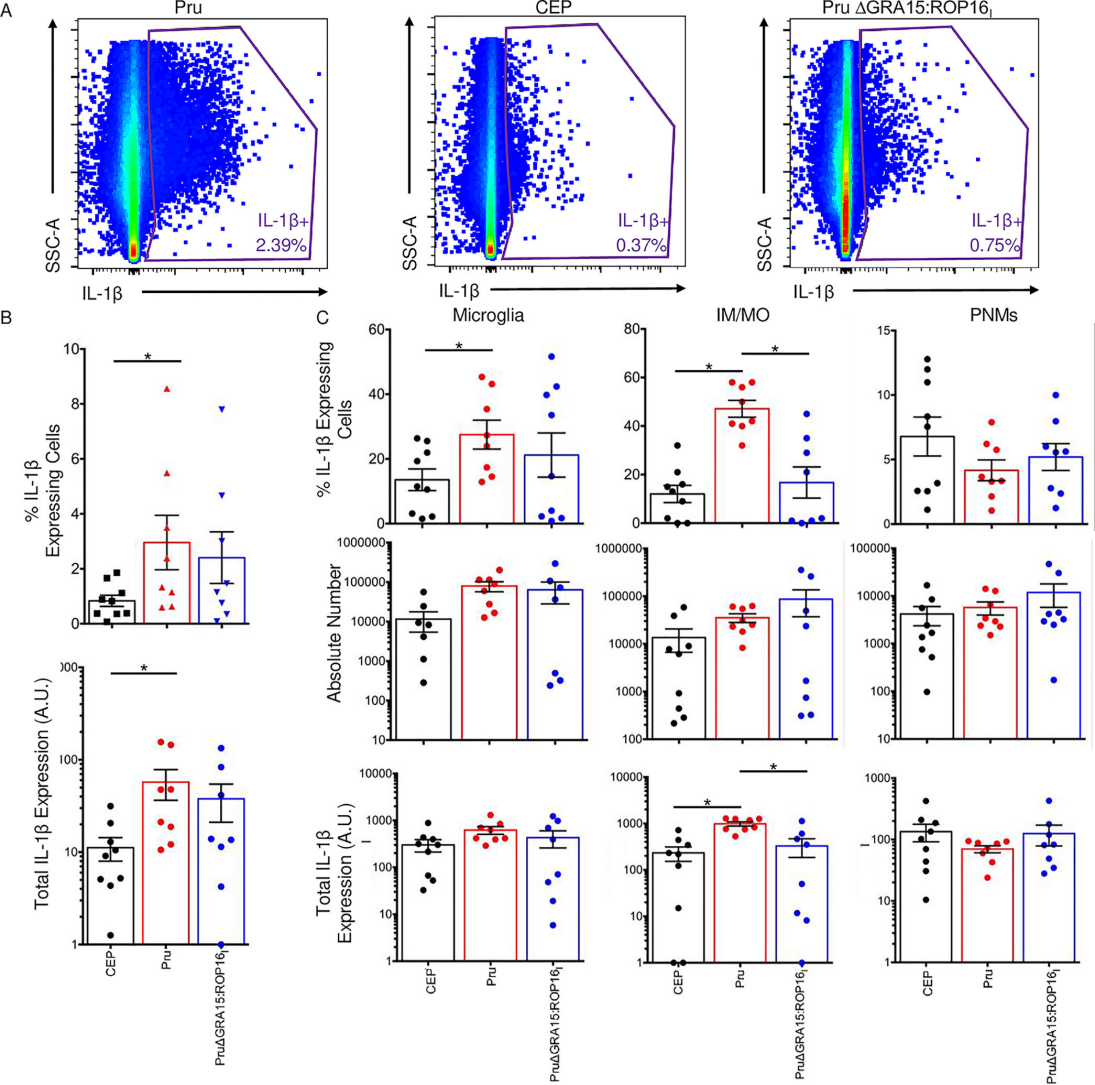
IL-1β protein expression in *Toxoplasma*-infected brains. (A) Brains from mock-infected mice (*n* = 3) or mice infected with CEP, Pru, or PruΔGRA15:ROP16_I_ (*n* = 8 to 9) were harvested 30 dpi and stained to detect pro-IL-1β expression by flow cytometry. IL-1β gate was determined from isotype-stained controls. (B) Quantification of the percentage of IL-1β-expressing cells and total IL-1β expression levels (% IL-1β-expressing cells; *, mean fluorescent intensity; A.U., arbitrary units). (C) IL-1β-expressing cells were gated to characterize the percent, number, and total IL-1β expression levels of IL-1β-expressing microglia, IM/MO, and polymorphonuclear (PMN) cells. Shown are means ± SEM of mice from 3 independent experiments. *, *P* ≤ 0.05; Mann-Whitney U test.

Next, IL-1β protein levels were examined by flow cytometry by staining single-cell suspensions from brains harvested from mock-infected mice or mice infected with Pru, CEP, or PruΔGRA15:ROP16_I_ strain parasites to detect pro-IL-1β. In brains infected with wild-type Pru strain parasites, 2.96 ± 0.99% (mean ± SEM) of the cells were IL-1β^+^, and this was significantly higher than those from CEP-infected mice (Fig. 6A and B). In addition, IL-1β expression levels were significantly higher in IL-1β-expressing cells from Pru-infected mice than from CEP-infected mice. Similarly, numbers and expression levels of IL-1β-expressing cells were comparable from mice infected with ME49 strain parasites (Fig. S4). Using a gating strategy similar to Fig. 4, we determined that the majority of IL-1β-expressing cells were myeloid derived (neutrophils, IM/MO, and microglia), and this was parasite strain independent (Fig. 6C). We next characterized the IL-1β-expressing cells, and numbers of each cell type were significantly lower in CEP strain-infected mice than Pru-infected mice but were comparable with GRA15-deficient parasites. But IL-1β expression levels were significantly higher in IM/MO from wild-type infected brains than from those infected with PruΔGRA15:ROP16_I_ strain parasites. Taken together, these data indicate that GRA15 regulates proinflammatory gene expression in brains of *Toxoplasma*-infected mice, including those involved in IL-1 signaling.

10.1128/mBio.01331-21.4 FIG S4 Comparison of IL-1β-expressing cells in type II *Toxoplasma*-infected brains. (A) Brains from mock (*n* = 3)-, ME49 (*n* = 9)-, or Pru (*n* = 8)-infected mice were harvested 30 dpi and stained to detect IL-1β expression by flow cytometry as described in Fig. 6. (B) Quantification of the percentage of IL-1β-expressing cells and total IL-1β expression levels (% of IL-1β-expressing cells; *, mean fluorescent intensity; A.U., arbitrary units). (C) Quantification of the percent, absolute number, and IL-1β total expression levels of IL-1β-expressing microglia, IM/MO, and PMNs. FACS plots are representative of 3 independent experiments. Data represents mean ± SEM. *, *P* ≤ 0.05; ns, not statistically different; Mann-Whitney U test. Download FIG S4, TIF file, 0.9 MB. Copyright © 2021 Glausen et al. 2021 Glausen et al. https://creativecommons.org/licenses/by/4.0/ This content is distributed under the terms of the Creative Commons Attribution 4.0 International license.

### PTZ-induced seizures in *Toxoplasma*-infected brains are dependent on IL-1 signaling.

**FIG 7:**
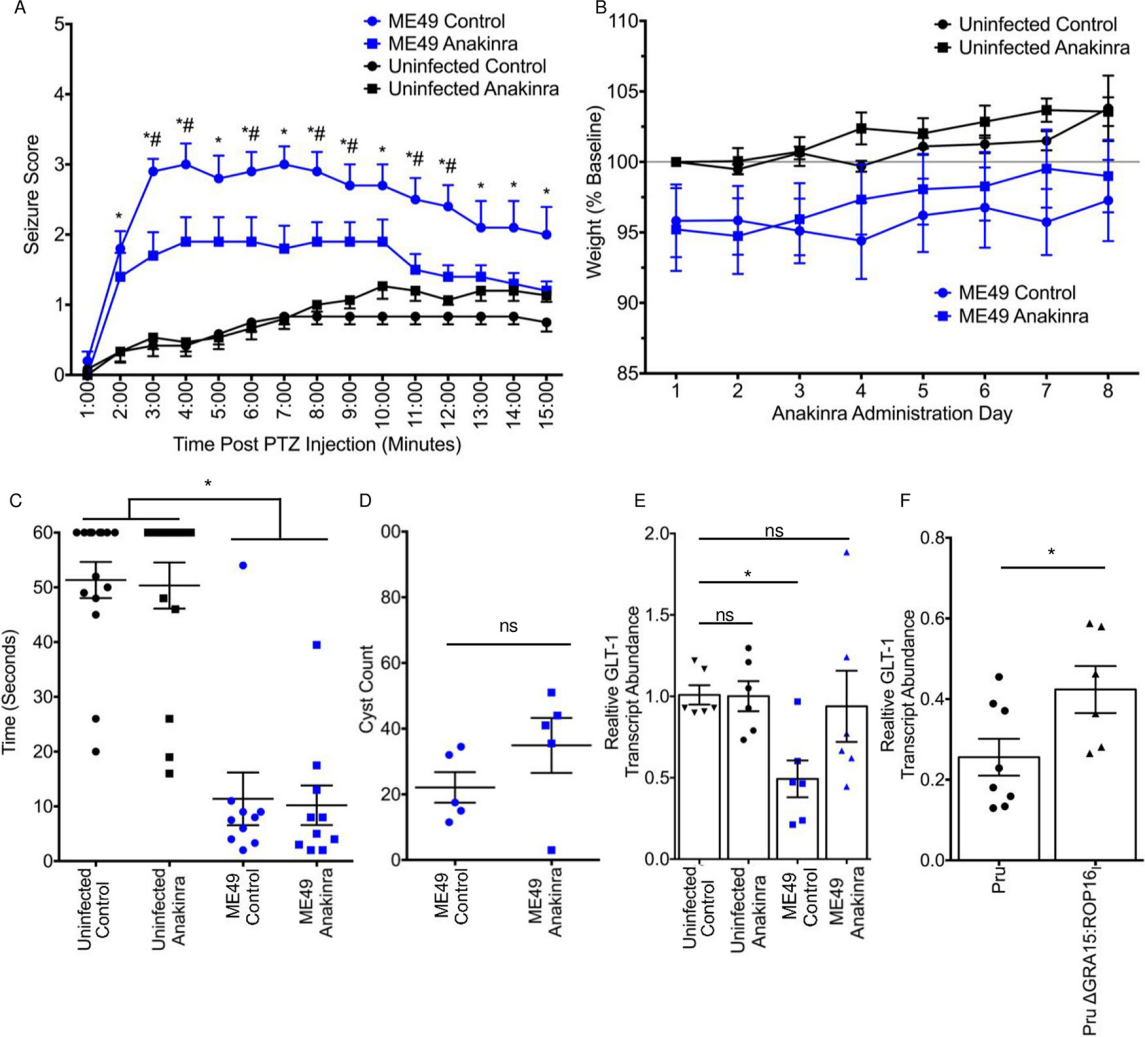
Anakinra reduces seizure severity in *Toxoplasma*-infected mice. (A) Mice were mock infected or infected with ME49 for 30 days and then received daily injections of anakinra (*n* = 10 to 13 per group). After 7 days, PTZ-evoked seizures were assessed. *, significant difference between ME49/PBS versus ME49/anakinra (*P* ≤ 0.05; Student's *t* test). (B) Mice were weighed daily during anakinra treatment and weight loss calculated based on weight on either the first day of infection or anakinra treatment (for mock infected). (C) After 7 days of treatment, mice were hang tested by placement on wire screen and hung upside down for up to 1 min. *, *P* ≤ 0.05; one-way analysis of variance (ANOVA). (D) Cysts were quantified in 12 to 16 nonconsecutive coronal brain sections between 0 and −3 mm from the anatomical landmark bregma (∼200 μm between sections); ns, not statistically different. Student's *t* test. (E) RT-PCR quantification of GLT-1 transcript abundance (normalized to mock-treated uninfected samples) in mock- and parasite-infected mice treated with anakinra or PBS. Data represent mean ± SEM from 3 independent experiments. *, *P* ≤ 0.05; one-way ANOVA. (F) RT-PCR quantification of GLT-1 transcript abundance (relative to uninfected controls) in Pru- and Pru ΔGRA15:ROP16_I_-infected mice. Data represents mean ± SEM from 3 independent experiments. *, *P* ≤ 0.05; Student's *t* test.

IL-1RA is an endogenous IL-1 receptor antagonist that binds the IL-1 receptor and blocks ligand-receptor interactions (42). Anakinra, a recombinant IL-1RA (43), was therefore used to test whether IL-1 signaling contributes to *Toxoplasma*-induced seizures. Mock- and ME49-infected mice were treated with daily intraperitoneal (i.p.) injections of anakinra (or phosphate-buffered saline [PBS] as a vehicle control) for 7 days starting at 30 days postinfection. The mice were then challenged with PTZ and seizure scores recorded. While PTZ evoked strong seizures in vehicle-treated ME49-infected mice, seizure severity was significantly reduced in the infected mice that were treated with anakinra (Fig. 7A). Reduced seizure severity in anakinra-treated mice was not a consequence of reduced morbidity, as no differences in weight loss or muscle fatigability (44) were observed (Fig. 7B and C). Similarly, anakinra had no significant effect on cyst burdens (Fig. 7D).

*Toxoplasma* is believed to trigger seizures through at least two mechanisms, mislocalization of the major GABA biosynthetic enzyme, GAD67, and decreased expression of the astrocytic glutamate transporter, GLT-1 (4, 5). We posited that IL-1 receptor signaling promotes seizure development by contributing to one or both of these phenotypes. Anakinra had no effect on GAD67 localization (Fig. S5A). This result is consistent with the finding that QTLs were not found on chromosome X after analyzing GAD67 mislocalization in mice infected with the 24 progeny strains (Fig. S5B and C).

10.1128/mBio.01331-21.5 FIG S5 GRA15/IL-1 signaling does not mediate GAD67 mislocalization. (A) Representative images of hippocampal GAD67 staining from mock- and parasite-infected mice treated with either PBS or anakinra. (B) Quantification of GAD67 mislocalization of hippocampal GAD67 staining from mock- and parasite-infected mice treated with either PBS or anakinra. *n* = 5 to 10 samples per group. Significant differences were not identified by one-way ANOVA. (C) QTL mapping of GAD67 mislocalization using the 24 progeny strains. Data represents mean ± SEM. *P* value of 0.05 equals LOD score of 2.08. Download FIG S5, TIF file, 1.3 MB. Copyright © 2021 Glausen et al. 2021 Glausen et al. https://creativecommons.org/licenses/by/4.0/ This content is distributed under the terms of the Creative Commons Attribution 4.0 International license.

Next, we tested whether decreased GLT-1 expression in *Toxoplasma*-infected brains is regulated by GRA15/IL-1 receptor signaling. First, mice infected with ME49 (or PBS as a vehicle control) were administered a 7-day anakinra regimen starting at day 30 postinfection. Mice were then euthanized, and RNA was isolated from bregma 0 to bregma −4 mm, which contains the hippocampus and cortex. Similar to previous work (5), GLT-1 transcript abundance was reduced ∼50% in ME49-infected mice (Fig. 7E). Anakinra significantly attenuated this decrease, and GLT-1 transcript levels were reduced by <20%. To test whether attenuation of decreased GLT-1 expression was GRA15 dependent, we repeated these experiments using Pru wild-type and PruΔGRA15:ROP16_I_ parasites. The data indicated that GLT-1 expression was significantly higher in brains from mice infected with GRA15-deficient parasites, although not as attenuated as in mice treated with anakinra (Fig. 7F). We did, however, fail to note significant increases in GLT-1 protein levels in either anakinra-treated mice or mice infected with GRA15-deficient parasites (not shown). Taken together, these data indicated that seizure susceptibility in *Toxoplasma*-infected mice is dependent on GRA15/IL-1 receptor signaling that is most likely independent of GAD67 mislocalization or decreased GLT-1 expression.

## DISCUSSION

Infections trigger encephalitic seizures due to a variety of reasons, including inflammation-induced alterations to synaptic structure, synaptic connectivity, blood-brain barrier integrity, and extracellular ionic environment (27, 45, 46). In most cases, the microbial-derived products that trigger seizures are unknown. Here, QTL mapping identified four loci in the *Toxoplasma* genome that correlate with PTZ-induced seizures in *Toxoplasma*-infected mice. In one locus, the polymorphic GRA15 gene was identified as an SLG since loss of GRA15 expression resulted in significantly reduced seizure responses and did so with minimal impact on morbidity or brain cyst burdens. GRA15 was originally identified as an activator of host proinflammatory gene expression during acute infections (14), and we demonstrate a similar role for GRA15 in brains of toxoplasmic encephalitic mice. Specifically, we showed that GRA15 mediates IL-1 pathway gene expression and that inhibiting IL-1 receptor signaling significantly reduced seizure severity in PTZ-treated mice. While IL-1 receptor signaling inhibition attenuated *Toxoplasma*-induced decreases in GLT-1 transcript abundance (5), it did not affect overall GLT-1 protein levels or GAD67 mislocalization, indicating that GRA15/IL-1 receptor signaling most likely promotes development of a proconvulsant microenvironment within the brain via other proteins and pathways.

Because *Toxoplasma* is haploid, QTL screening has emerged as a powerful genetic screening approach to study *Toxoplasma* phenotypes using a limited number of progeny (10–14, 47–49). In our study, the identified SLG loci did not reach statistical significance, and we are therefore cautious in their interpretation. But confidence is bolstered with GRA15 serving as a proof-of-principle finding. A QTL simulation of virulence data from reference 11 using only the progeny strains used in this study identified peaks corresponding to 4 of the 5 VIR loci. Importantly, these peaks were also below statistical significance, indicating that the inability for SLG loci to reach statistical significance was due to the limited number of progeny tested. In addition, QTL screening revealed that seizure and cyst burden phenotypes were not linked. This was expected because, while cysts may impact some behavioral changes (50), *Toxoplasma* cysts likely have a limited role in inducing toxoplasmic encephalitic seizures for several reasons, including (i) subclinical and clinical (e.g., seizures) toxoplasmic encephalitis develop in response to proliferating tachyzoites (23, 51), (ii) bradyzoites are considered significantly less immunogenic/inflammatory than tachyzoites, and (iii) chronically infected individuals show little signs of neuronal inflammation (52, 53). In contrast, we did find an association (but not absolute cosegregation) of seizure and morbidity phenotypes, although it remains unclear whether weight loss due to inappetence is linked to an altered neuronal signaling pathway involved in feeding or is a general manifestation of toxoplasmosis.

Besides GRA15, two other QTLs from reference 11 mapped to a region near an SLG locus. SLG1 maps to a region on chromosome XII that contains ROP5, which is the master regulator of *Toxoplasma* virulence (12, 13). Although seizure severity and morbidity phenotypes are linked, it is important to note that virulent ROP5 alleles do not cosegregate with morbidity, and it is possible that other gene(s) located within SLG1 are involved in seizures. Likewise, while SLG4 contains GRA15, other seizure-linked genes may be located within SLG4. Thus, future work will focus on identifying additional SLGs that we predict function to either promote parasite dissemination to the brain, promote inflammatory responses, or directly impact neuronal function and synaptic signaling. These genes may include those that promote tachyzoite proliferation, which is an important parameter to measure. But it is important to note that seizure susceptibility does not necessarily correlate with tachyzoite burdens within the brain. This is best exemplified by the finding that type III strain parasites do not develop seizures at 21 days postinfection (dpi) (but type II strains do), which is a time point that brain tachyzoite burdens are similar between the strains, and brains infected with type III strain parasites have increased inflammation (19).

Seizures develop due to a disbalance of excitatory and inhibitory neurotransmission, both of which are impacted in *Toxoplasma*-infected mice (4, 5, 54). For example, we reported that *Toxoplasma* infection leads to GAD67 mislocalization (4). Similar to morbidity, we found a correlation between GAD67 mislocalization and seizure susceptibility phenotypes (*P* = 0.45; McNemar chi-square test), but GRA15 was not found to be associated with GAD67 mislocalization QTLs (Table S2 in the supplemental material; Fig. S5). GRA15/IL-1 receptor signaling decreased GLT-1 transcript abundance, which is consistent with the finding that IL-1 signaling represses GLT-1 expression (55). We were not, however, able to detect changes in GLT-1 protein abundance, indicating that the effect of GRA15/IL-1 receptor signaling on seizures is most likely independent of GLT-1. It is possible that that relatively minor changes in GLT-1 protein abundance may be sufficient to impact seizure susceptibility in *Toxoplasma*-infected mice. But the most likely interpretation of these data is that GAD67 mislocalization and GLT-1 repression occur independently of GRA15/IL-1 receptor signaling, but the three culminate to trigger a single phenotype—seizures.

GRA15 is a dense granule protein that is secreted into the infected host cell and associates with the parasitophorous vacuole, where it engages host TRAF proteins to activate NF-κB and upregulate the expression of target genes, including those involved in IL-1 signaling (14, 56). Relative to bradyzoites, GRA15 is significantly more highly expressed by tachyzoites in the brain (29–31). Here, we demonstrated that GRA15 expression correlates with enhanced expression by IM/MO and microglia of many of these genes, including IL-1β. However, it remains unclear how GRA15 is triggering this response within the brain. One possibility is that GRA15 is secreted directly into infected IM/MO or microglia following spontaneous reactivation of encysted parasites, leading to upregulation of IL-1 ligands as well as activation of the machinery required to process and release them. But we discount that possibility since others demonstrated that neurons are the primary targets for *Toxoplasma* infection in the brain (57). On the other hand, we favor two other nonmutually exclusive hypotheses. First, GRA15 expression may allow for increased release of pathogen-associated molecular patterns (PAMPs) and danger-associated molecular patterns (DAMPs) that would bind to and activate IM/MO. Since our data revealed that GRA15 did not significantly impact numbers of IM/MO cells, this enhanced replication would likely have more discrete effects on inflammatory responses such as enhanced IL-1α/IL-1β expression and secretion. Second, GRA15 may trigger infected neurons to secrete chemokines and cytokines that would stimulate IM/MO and microglia recruitment and their subsequent release of the IL-1 receptor ligands IL-1α and IL-1β, both of which are expressed in *Toxoplasma*-infected brains (58). The finding that infection stimulates microglial ensheathment of neuronal somas, leading them to phagocytose and/or displace inhibitory perisomatic synapses, supports such a model (54). Finally, it is possible that, similar to ROP16, GRA15 expression early during infection in the periphery may impact neuronal inflammation (19). However, the ability for anakinra to be applied 4 weeks postinfection and inhibit seizures supports a model in which seizures are induced by GRA15 expression within the brain.

IL-1 receptor expression in the brain is widespread and includes endothelial cells, neurons, and astrocytes (58–63). However, it is currently unclear which cell(s) is/are expressing the IL-1 receptor to regulate seizure activity. Recently, it was reported that IL-1α release by microglia triggered IL-1 receptor signaling on endothelial cells, which altered their adhesion molecule expression to enable inflammatory cell recruitment to the brain (58). But we found no significant role for GRA15 in regulating inflammatory cell recruitment to the brain but, instead, found that it impacted expression of IL-1 associated genes. And while genetic ablation of IL-1 signaling impacts neuronal immune responses against *Toxoplasma* (58), short-term antagonism of this pathway in established infections has more discrete impacts on neuronal function. Rather than a discrepancy between the two studies, we believe that these data highlight the interplay between distinct *Toxoplasma* effectors and their host to fine-tune anti-*Toxoplasma* immune responses. Addressing this will require identifying which specific IL-1 receptor ligand(s) is involved in seizure development during toxoplasmic encephalitis as well as defining which cells are the source(s) for the cytokine(s). Microglia, which we reported displaces inhibitory GABAergic synapses in *Toxoplasma*-infected brains (64), are likely sources. But whether microglia are monolithic or whether there are functionally distinct microglial populations—e.g., that express IL-1 ligands and those that displace inhibitory synapse—remains to be resolved.

In summary, we have identified a role for GRA15 besides its well-ascribed function during the acute phase of *Toxoplasma* infections. We demonstrated that during toxoplasmic encephalitis, GRA15 promotes a proconvulsant state by increasing IL-1-receptor signaling. Moreover, we show that inhibiting IL-1 signaling significantly reduced seizure severity, suggesting a novel therapeutic approach toward treating seizures in toxoplasmic encephalitic patients as well as those suffering from other infection-induced seizures.

## MATERIALS AND METHODS

### Parasites.

The following *Toxoplasma* strains were used: ME49, Pru (clone PA7 [17]), CEP, PruΔGRA15 (14), PruΔGRA15:ROP16_I_ (1B7 [17]), and 24 progeny strains from 2 independent crosses between ME49 and CEP (21, 65). Parasites were routinely cultured on human foreskin fibroblasts 1 (HFF-1) at 37°C in Dulbecco’s modified Eagle’s medium (DMEM) supplemented with 10% heat-inactivated fetal calf serum (FBS), 1% l-glutamine, and 1% penicillin-streptomycin as previously described (66). Parasites and HFFs were routinely tested for *Mycoplasma* (MycoAlert; Lonza, Basal, Switzerland) and found to be negative. Unless otherwise stated, all chemicals were purchased from Sigma (St. Louis, MO) or VWR (Radnor, PA) as the highest grade available.

### Mouse infections and treatments.

Eight- to ten-week-old female C57BL/6J mice (Jackson Laboratory, Bar Harbor, ME) were intraperitoneally (i.p.) infected with tachyzoites or tissue cysts resuspended in Hanks balanced salt solution (HBSS) or PBS, respectively. Mice were monitored daily for weight, posture, and activity level over the course of infection. Mice with significant weight loss (>15%) were provided moist chow and supplemental diet gel. PTZ assays were performed and seizures scored as described (4). Briefly, mice were injected i.p. with PTZ (40 mg/kg in PBS) and scored every 30 s for 15 min using the following scoring criteria: 0, normal activity; 1, lowered prostrate and reduced movement; 2, partial clonus; 3, generalized clonus; 4, tonic-clonic seizures; and 5, death. Anakinra (Sobi, Stockholm, Sweden) was injected i.p. (25 mg/kg in 200 μl or PBS as vehicle control) for 7 days.

### Quantitative trait locus analysis.

QTL analysis was performed using genetic marker data from *Toxoplasma* Genome Map Database (http://toxomap.wustl.edu/). Data from both crosses were downloaded and compiled into one file of 134 genetic markers for QTL analysis based on binarized seizure phenotype using R/qtl (67, 68). In short, phenotype and genetic data were loaded into R/qtl. The genetic map was calculated using the haldane function, and QTL was performed using multiple imputation. LOD was calculated using 1,000 permutations.

### Immunohistochemistry.

Mice were anesthetized with ketamine (10 mg/kg) and exsanguinated with sterile PBS and then perfused with 4% paraformaldehyde in PBS. Brains were harvested and cryoprotected in 30% sucrose, and 20-μm-thick sections were prepared. Samples were incubated in blocking buffer (5% goat serum, 2.5% bovine serum albumin, and 0.1% Triton X-100) for 1 h and stained using the antibodies listed in Table S5 or with Dolichos biflorus agglutinin (DBA; Vector Laboratories, Burlingame, CA) for 2 h to detect tissue cysts. Samples were then repeatedly washed in PBS and coverslips mounted using DAPI (4′,6-diamidino-2-phenylindole) Vectashield mounting media (Vector Laboratories). Cysts were enumerated by analysis of 12 to 16 coronal brain sections between −3 to 0 mm from landmark bregma, and an ME49 phenotype was denoted as observing >5 cysts of at least 5 μm in diameter (CEP cysts were fewer and smaller [69]). GAD67 immunoreactivity was quantified by calculating the ratio of mean fluorescent intensity (determined using ImageJ) in the stratum pyramidalis to surrounding hippocampal layers (stratum radiatum and stratum oriens). DAPI was used to differentiate between neuronal layers. Note that this was only performed for low-dose infections.

10.1128/mBio.01331-21.10 TABLE S5 List of antibodies used in this study. Download Table S5, XLSX file, 0.01 MB. Copyright © 2021 Glausen et al. 2021 Glausen et al. https://creativecommons.org/licenses/by/4.0/ This content is distributed under the terms of the Creative Commons Attribution 4.0 International license.

### Flow cytometry.

Following euthanasia by CO_2_ asphyxiation and perfusion with ice-cold PBS, single-cell brain suspensions were prepared by incubating minced tissues in digestion media (RPMI 1640, 1% penicillin-streptomycin, 1% l-glutamine, 0.1% beta-mercaptoethanol [β-ME], 25 mM HEPES, 150 μg/ml DNase, and 59 μg/ml Liberase TL). Homogenates were passed through a 70-μm filter, washed in PBS, and centrifuged through a Percoll gradient (37.5% Percoll in HBSS) at 650 × *g* for 20 min. The pellet was resuspended in RPMI 1640 supplemented with 10% FBS, 1% penicillin-streptomycin, 0.1% β-ME, 25 mM HEPES (pH 7.0). Cells were stained with Live/Dead fixable aqua (Thermo Fisher, Waltham, MA) or with antibodies against cell surface markers (Table S5). Intracellular antigens were detected by fixing and permeabilizing cells overnight in FOXP3 transcription factor staining buffer set (Thermo Fisher) followed by antibody incubation. Samples were washed and resuspended in fluorescence-activated cell sorter (FACS) buffer (0.5 mM EDTA, 5% FBS, 0.001% sodium azide, and 1× PBS). Data were acquired using the BD LSRFortessa cell analyzer and analyzed using FlowJo version 10.0.8 (TreeStar, Ashland, OR). IL-1β total expression was calculated as % IL-1β^+^ cells × mean fluorescence intensity of population (70).

### RNA-seq.

RNA was prepared from flash-frozen hippocampi using the Absolutely RNA purification kit (Agilent, Santa Clara, CA). RNA libraries were prepared from 1 μg total RNA using the Illumina Stranded TruSeq RNA library prep kit (Illumina, San Diego, CA). Final libraries were pooled to 10 nM, denatured, and loaded onto a HiSeq 2500 in high-output mode with PE100 for sequencing reads. Normalized counts were calculated for each gene. Pathway analysis was performed using DAVID (71, 72), and data visualization was performed using the R gplots package (68).

### RT-PCR.

Total RNA was converted to cDNA using Superscript III RT (Thermo Fisher) and target genes amplified using Power Sybr green PCR master mix (Thermo Fisher). Target expression levels were determined using the threshold cycle (2^−ΔΔ^*^CT^*) method (66). Primer sets used include β-actin, 5′-GACGGCCAGGTCATCACTATTG-3′ and 3′-CCACAGGATTCCATACCCAAGA-5′; IL-1β, 5′-TGTGTGACGTTCCCATTAG-3′ and 3′-CCAAGGCCACAGGTATTT-3′; and GLT-1 (EAAT-2), 5′-CTCTCACTGACTGTGTTTGG-3′ and 5′-GGGAAGGCTATCAACATGAC-3′.

### Hang test.

Assays were performed as described (44). Briefly, mice were placed on a mesh sheet of wire with 1- by 1-cm holes that was inverted above a padded container for 60 s, at which time the trial ended.

### Statistics.

All statistical assays were performed using GraphPad Prism v6.0c (GraphPad, La Jolla, Ca.).

### Study approvals.

All animal procedures were approved by the University at Buffalo Institutional Animal Care and Use Committee (MIC23035Y).
