# Supplementary figures and images for: The Toxoplasma Polymorphic Effector GRA15 Mediates Seizure Induction by Modulating Interleukin-1 Signaling in the Brain
Source: mBio. 2021 Jun 22;12(3):e01331-21. doi: 10.1128/mBio.01331-21 (PMC8262954; doi:10.1128/mBio.01331-21)

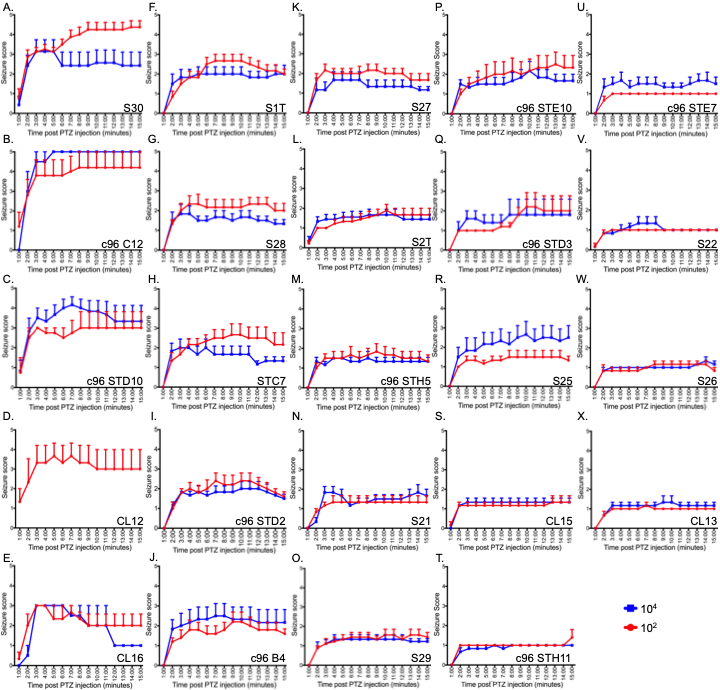

Supplement: FIG S1 [file mbio.01331-21-sf001.tif]

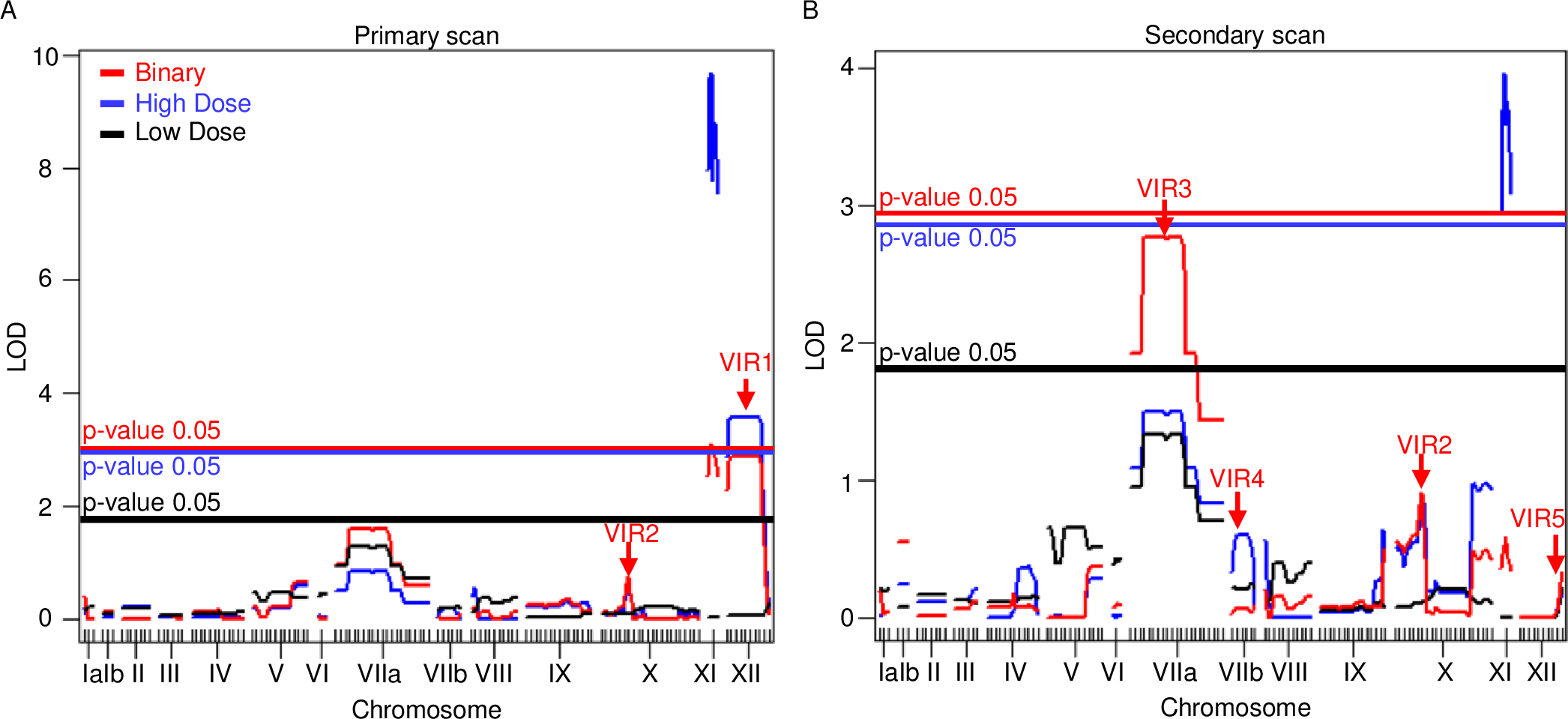

Supplement: FIG S2 [file mbio.01331-21-sf002.tif]

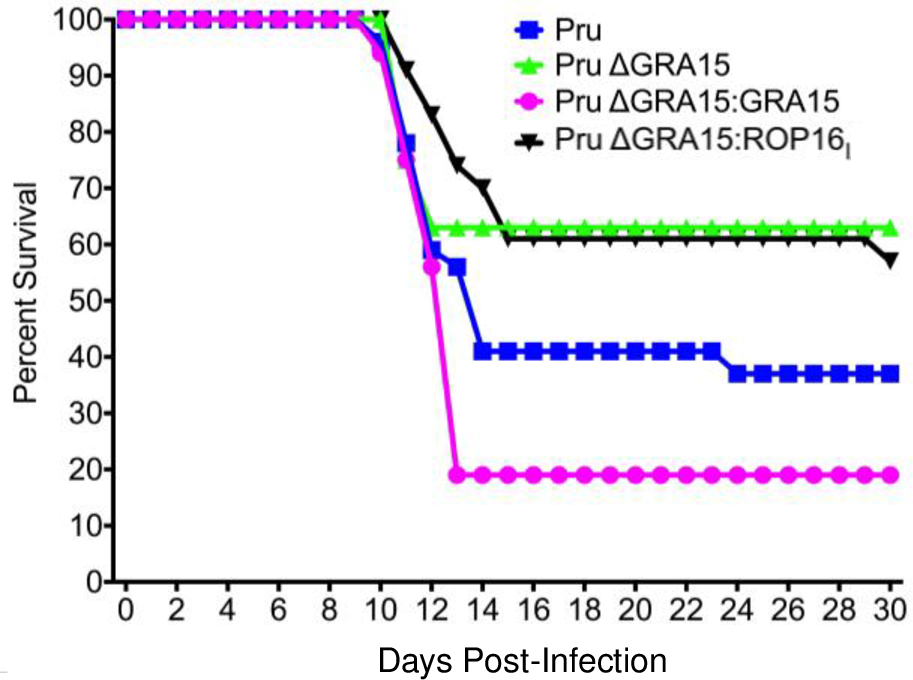

Supplement: FIG S3 [file mbio.01331-21-sf003.tif]

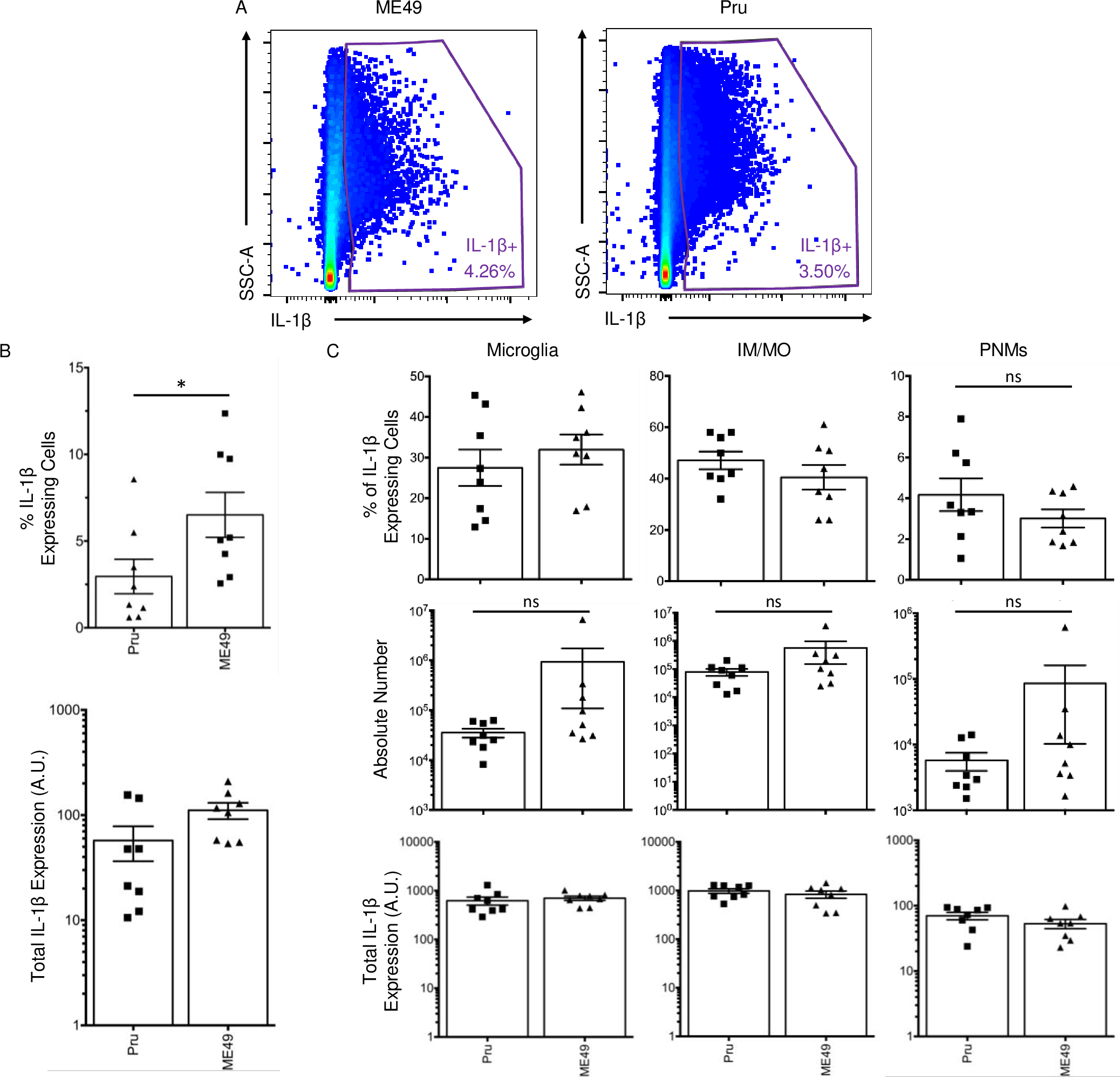

Supplement: FIG S4 [file mbio.01331-21-sf004.tif]

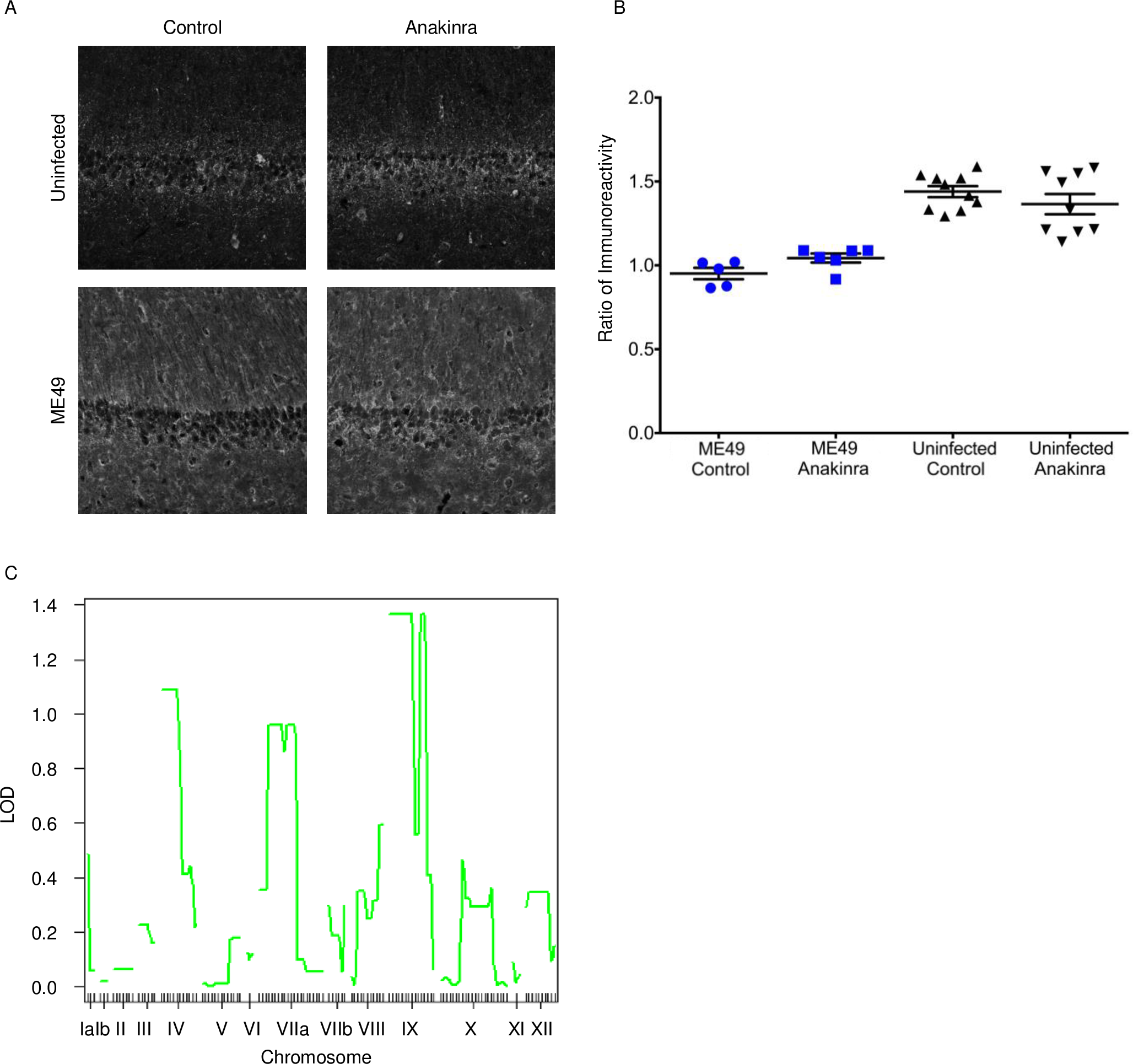

Supplement: FIG S5 [file mbio.01331-21-sf005.tif]
